# Extracorporeal Femoro-Carotid Shunt for Transcarotid Transcatheter Aortic Valve Replacement

**DOI:** 10.1016/j.jaccas.2024.102371

**Published:** 2024-05-13

**Authors:** Robert Semco, Ross G. McFall, Jay Khambhati, Pinak Shah, David Gross, Piotr S. Sobieszczyk, Ashraf A. Sabe

**Affiliations:** a Harvard Medical School, Boston, Massachusetts, USA; b Division of Cardiac Surgery, Department of Surgery, Brigham and Women’s Hospital, Harvard Medical School, Boston, Massachusetts, USA; c Division of Cardiovascular Medicine, Department of Medicine, Brigham and Women’s Hospital, Harvard Medical School, Boston, Massachusetts, USA

**Keywords:** acute heart failure, aortic valve, femoro-carotid shunt, transcarotid, transcatheter aortic valve replacement, valve replacement

## Abstract

Transcatheter aortic valve replacement may be performed with a transcarotid approach when peripheral vascular disease is prohibitive for transfemoral access. In this case, a patient who presented in cardiogenic shock secondary to severe aortic stenosis developed electroencephalographic changes during transcarotid TAVR. A temporary extracorporeal femoro-carotid shunt permitted successful TAVR.

## Graphical Abstract

**Figure:**
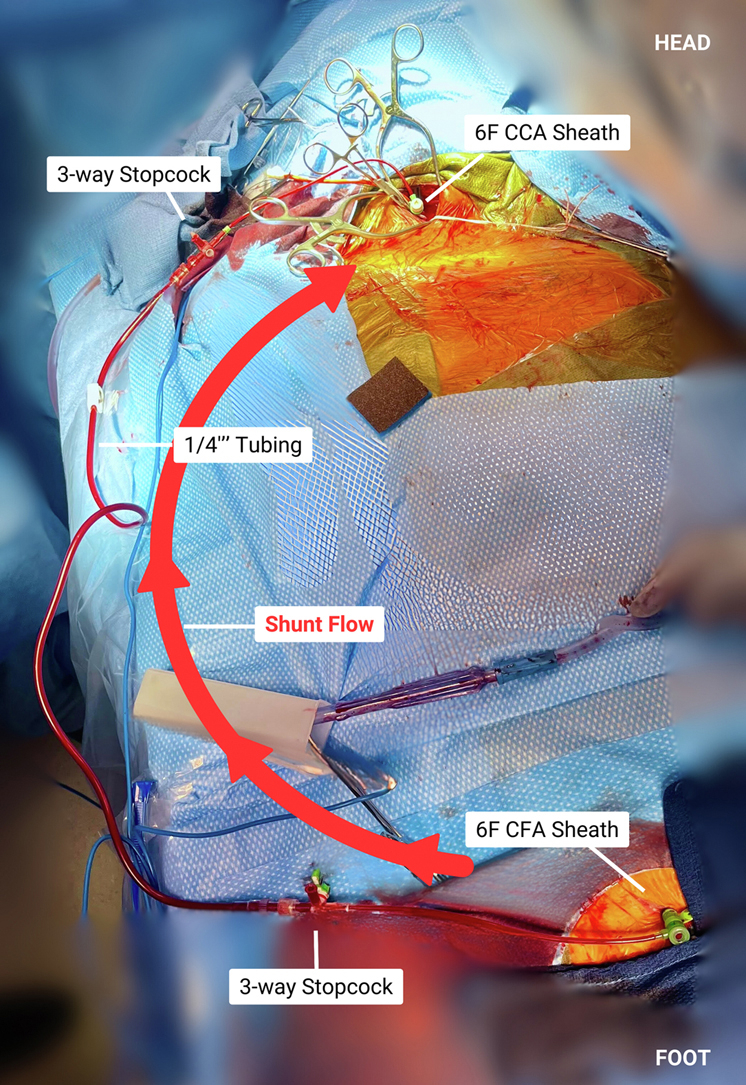

Three days before a planned transcatheter aortic valve replacement (TAVR), a 70-year-old man presented to the emergency department after his preoperative laboratory results demonstrated a hematocrit of 22.1%. Upon presentation, he reported weeks of worsening dyspnea on exertion, orthopnea, and paroxysmal nocturnal dyspnea. His blood pressure was 109/56 mm Hg, his heart rate was 74 beats/min, and he required 2 L of oxygen by nasal cannula. On physical examination, he had increased work of breathing, bilateral coarse breath sounds, a Class II/VI late peaking systolic heart murmur, jugular venous distension, and lower extremity edema.

**Learning Objectives**

• To be able to establish a femoro-carotid shunt using the Seldinger technique when performing transcarotid TAVR.
• To understand the importance of electroencephalogram monitoring and contingency planning in the completion of a safe transcarotid TAVR.

## Past Medical History

The patient had received a diagnosis of severe aortic stenosis 1 month prior, and he had since completed his preoperative evaluation for aortic valve replacement. He also had a history of insulin-dependent type 2 diabetes mellitus, stage IIIb chronic kidney disease, peripheral artery disease (status post right superficial femoral artery stenting), chronic left common iliac artery dissection, and coronary artery disease (status post quadruple coronary artery bypass grafting) complicated by stroke without residual deficits. His home antiplatelet regimen consisted of aspirin. Owing to his multiple comorbidities and prior cardiac surgery, he was offered TAVR, which was to be performed with a transcarotid approach because of his heavily diseased iliofemoral systems and abdominal aorta, presence of a left internal mammary artery, and tortuosity disfavoring an approach from the right axillary artery.

## Differential Diagnoses

The initial differential diagnoses included acute heart failure due to severe aortic stenosis, anemia of chronic disease, upper gastrointestinal bleed, Heyde’s syndrome, or pneumonia.

## Investigations

The laboratory results were notable for leukocytosis to 17.4 K/μL, hyperkalemia to 6.1 mmol/L, serum urea nitrogen/creatinine of 107 mg/dL/3.39 mg/dL, and elevated lactate to 7.5 mmol/L. Transthoracic echocardiography demonstrated a left ventricular ejection fraction of 35% and very severe aortic stenosis with a peak ejection velocity of 5.1 m/s, mean pressure gradient of 75 mm Hg, and an average valve area of 0.6 cm^2^. Preoperative evaluation with computed tomography had revealed moderate stenosis of the right common iliac artery, in-stent restenosis of the right superficial femoral artery, and stable focal dissection of the left common iliac artery. Duplex ultrasonography of the bilateral carotid arteries demonstrated 50% to 69% stenosis of the right internal carotid artery but <50% stenosis in the right common carotid artery (CCA) and the left internal and common carotid arteries.

## Management

The patient was found to be in cardiogenic shock resulting from severe aortic stenosis and was admitted to the cardiac intensive care unit, where he required vasopressors to maintain mean arterial pressure >65 mm Hg. He received 3 U of packed red blood cells and was medically optimized before TAVR with lactate improved to 1.0 mmol/L preoperatively while he was receiving dobutamine.

On hospital day 3, the patient underwent transcarotid TAVR with intraoperative electroencephalogram (EEG) monitoring. Given the significant disease in the right internal carotid artery (ICA), the right CCA was selected as the access to maintain cerebral perfusion through the patent left ICA, theoretically decreasing the risk of ischemia with clamping.^1^ He was positioned supine, induced under general anesthesia, and prepared and draped to provide exposure of the anterior part of the neck, right side of the chest, right upper extremity, and proximal right lower extremity. Access was obtained with a 6-F left radial artery sheath and a 6-F right common femoral vein sheath.

**Figure 1:**
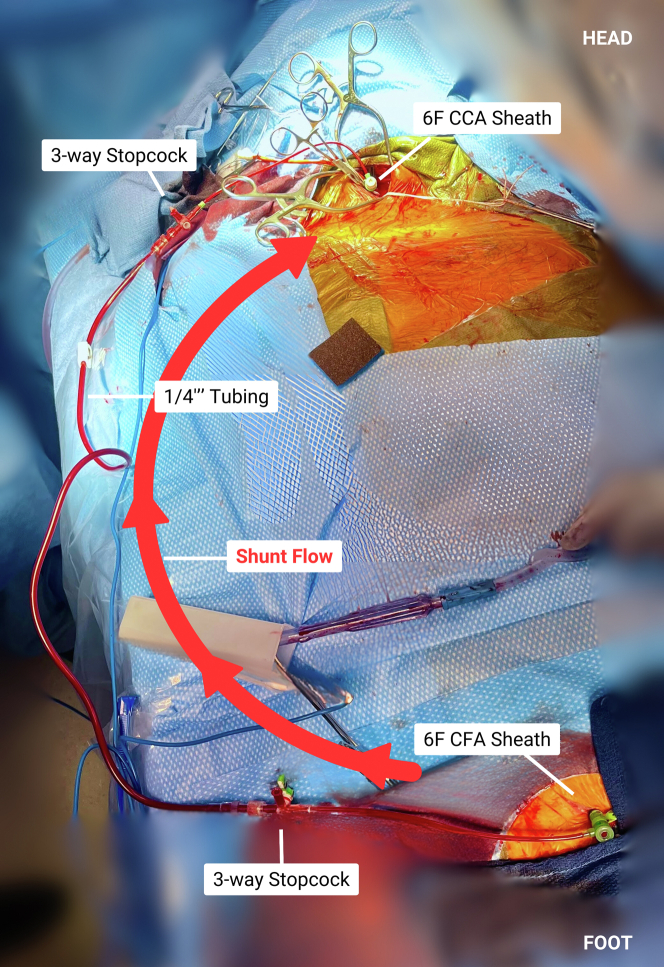
Femoro-Carotid Shunt in Place CCA = common carotid artery; CFA = common femoral artery.

**Figure 2:**
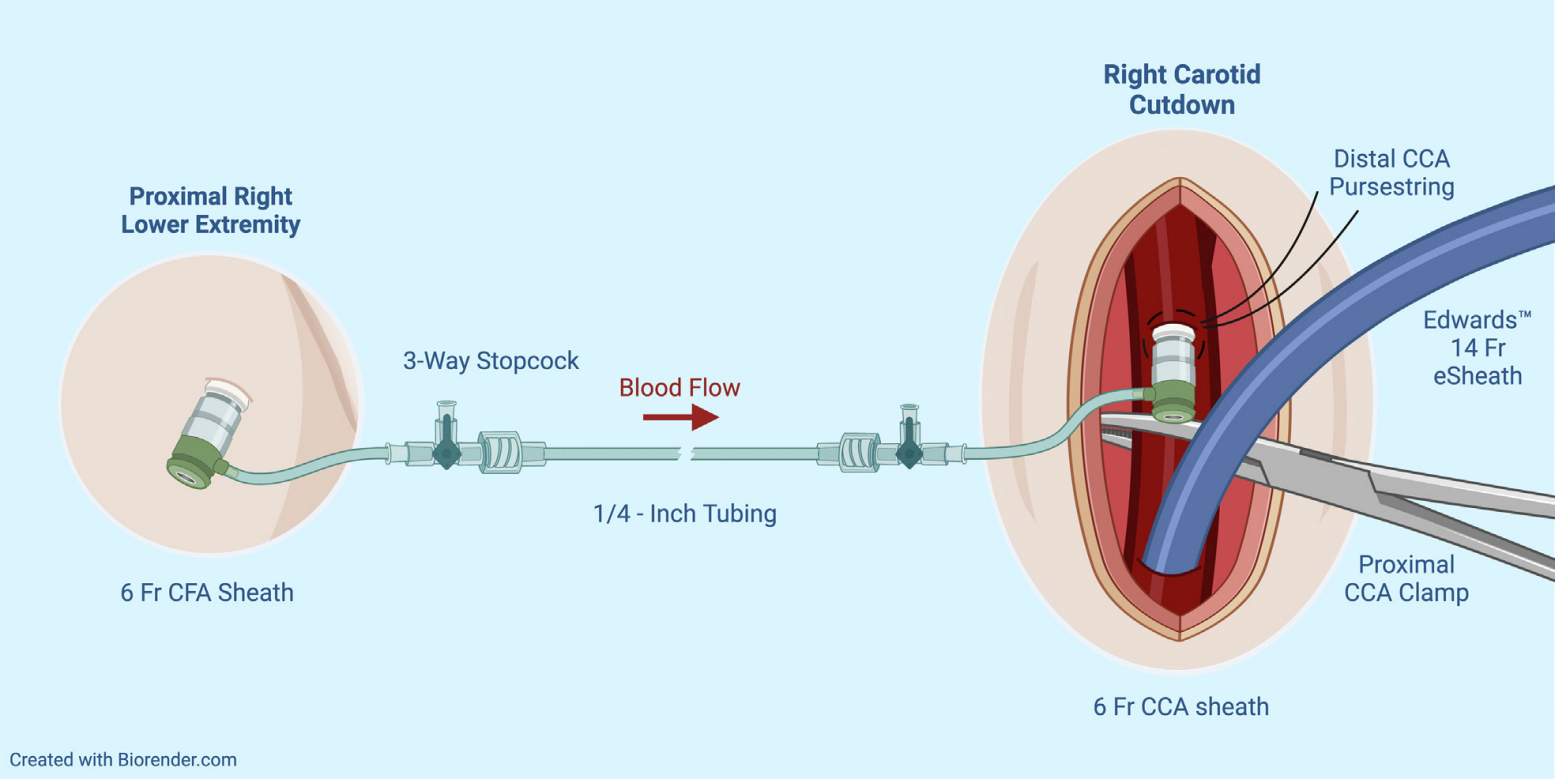
Femoro-Carotid Shunt Schematic Abbreviations as in Figure 1.

An incision was made at the medial border of the right sternocleidomastoid and was carried down to expose the right CCA. The patient was given systemic heparin for an activated clotting time goal >250 seconds. The right CCA was subsequently test-clamped, which resulted in attenuation of right-sided EEG amplitude. These EEG changes persisted, despite elevating mean arterial pressures to 80 to 90 mm Hg using 4 pressors, as well as a measured stump pressure measuring >40 mm Hg. Given these EEG findings, we elected to create a shunt between the right common femoral artery (CFA) and right CCA, as seen in Figures 1 and 2.

The right CFA was accessed under ultrasound guidance, and a standard 6-F sheath was inserted, with the patient still under full heparinization. Using 6-0 Prolene, a pursestring was placed in the CCA, distal to the proposed clamp site. In the pursestring, the CCA was accessed directly using a needle and wire, and a 6-F short bright-tipped sheath was inserted via the Seldinger technique and connected to a ¼-inch tube. The carotid sheath was secured in place with a stitch to the edge of the wound to prevent movement and unintentional removal. The ¼-inch tube was connected to the 6-F right CFA sheath, and using the sheath’s 3-way stopcock, careful de-airing was performed. With the shunt in place and open, the right CCA was clamped without EEG changes for >5 minutes of monitoring. At that time, the decision was made to proceed with the TAVR.

Access to the CCA was obtained with a Cook needle, and a 10-F sheath was subsequently introduced. An AL1 catheter and straight wire were used to cross the aortic valve and were exchanged for a pigtail catheter. The tract was dilated, a 14-F expandible sheath was placed, and a 26-mm Sapien 3 Ultra valve (Edwards Lifesciences) was deployed under rapid pacing. After deployment, an angiogram and a transesophageal echocardiogram demonstrated a well-positioned and well-seated valve without paravalvular leak and no pericardial effusion. The right CCA was flushed distally and proximally and was closed in layers. The carotid sheath for the shunt was removed, and the pursestring was tied, with good hemostasis. The patient was extubated in the operating room and was neurologically intact at completion of the procedure.

## Discussion

This case describes the selective reversal of cerebral hypoperfusion via a temporary femoro-carotid extracorporeal shunt fashioned from 2 6-F sheaths, placed using the Seldinger technique, to permit transcarotid TAVR. This shunt was necessary for this patient, who was in cardiogenic shock, had no other approach to emergently convert to for TAVR, and who was not a candidate for surgery. The novel application of this simple, easily adaptable technique permitted the safe completion of an essential valve replacement.

The use of the Seldinger technique to create a femoro-carotid shunt has been previously described in the performance of carotid endarterectomies, where 8-F catheters were used to cannulate the femoral and carotid arteries.^2^ However, a 1-way roller pump was used to ensure flow from the femoral to the carotid catheter, whereas in the present case, an effective shunt was established without a pump, significantly simplifying the system. Other shunts have been used to temporarily re-establish extremity perfusion after traumatic injury, and although these shunts also did not use a pump, they were used to replicate normal anatomy.^3^ Distal femoral arterial perfuser sheaths are commonly performed upon placement of cannulas for extracorporeal membrane oxygenation and occasionally upon placement of Impella repositioning sheaths that are occlusive in the femoral system.^4^

To our knowledge, a temporary femoro-carotid shunt as described here has not been previously reported in the performance of transcarotid TAVR. Other shunts have been described for this purpose, although with differing techniques. In 1 case series, femoro-carotid shunts were routinely used for all patients undergoing transcarotid TAVR,^5^ where the femoral artery was percutaneously cannulated with a 16-F cannula and was then connected to a 14/15 Sundt shunt (Integra Lifesciences) placed in the CCA via arteriotomy. In another series, the CFA was cannulated with an 8-F sheath and connected to a carotid Polyshunt (Perouse Medical) placed within the CCA, also via arteriotomy, in patients with loss of consciousness during carotid test clamping.^6^ In the present case, however, the femoro-carotid shunt was established only when the patient demonstrated signs of cerebral hypoperfusion on EEG, and the CCA was cannulated via the Seldinger technique, avoiding arteriotomy and simplifying closure. In another case series of transcarotid TAVR, 2 patients received femoro-carotid shunts, but the technique used was not described.^7^

## Follow-Up

The patient’s postoperative stay was without complication, and he was subsequently weaned off all vasopressors at the case conclusion and oxygen. At the time of discharge, the patient was feeling well and ambulating without difficulty.

## Conclusions

To our knowledge, this is the first case report to describe the creation of a temporary extracorporeal femoro-carotid sheath shunt using the Seldinger technique during transcarotid TAVR. It was safe and effective in permitting life-saving valve replacement when no other endovascular approach was available. This technique can be considered alongside the other methods described here when a temporary femoro-carotid shunt is required.

## Funding Support and Author Disclosures

Dr Gross has been a consultant and proctor for Edwards Lifesciences. Dr Shah has been a proctor for Edwards Lifesciences; has received educational grants from Edwards Lifesciences, Medtronic, and Abbott; and has been an advisory board member for Xenter. All other authors have reported that they have no relationships relevant to the contents of this paper to disclose.

## References

[1] Allen K.B., Chhatriwalla A.K. (2023). The 10 commandments of transcarotid transcatheter aortic valve replacement. Innovations (Phila).

[2] Fachinetti P., Bellocchi S., Ramponi G., Sardella M., Dorizzi A. (2001). Carotid endarterectomy: a new technique replacing internal shunts. Acta Neurochir (Wien).

[3] Antunes L.F., Botelho M., Fonseca M. (2022). Extracorporeal sheath shunt technique in trauma: a different vascular shunt in civilian trauma. Vascular.

[4] Makdisi G., Makdisi T., Wang I.W. (2017). Use of distal perfusion in peripheral extracorporeal membrane oxygenation. Ann Transl Med.

[5] Thourani V.H., Li C., Devireddy C. (2015). High-risk patients with inoperative aortic stenosis: use of transapical, transaortic, and transcarotid techniques. Ann Thorac Surg.

[6] Debry N., Delhaye C., Azmoun A. (2016). Transcarotid transcatheter aortic valve replacement. J Am Coll Cardiol Int.

[7] Colegrave N., Mascitti P., Zannis K. (2021). Ultrasound-guided intermediate cervical plexus block for transcarotid transcatheter aortic valve replacement. J Cardiothorac Vasc Anesth.

